# Differences in glycemic control across world regions: a *post-hoc* analysis in patients with type 2 diabetes mellitus on dual antidiabetes drug therapy

**DOI:** 10.1038/nutd.2016.25

**Published:** 2016-07-04

**Authors:** H Brath, P M Paldánius, G Bader, W M Kolaczynski, P M Nilsson

**Affiliations:** 1 Diabetes Outpatient Clinic, Health Centre South, Vienna, Austria; 2 Novartis Pharma AG, Basel, Switzerland; 3 Department of Clinical Sciences, Skåne University Hospital, Malmö, Sweden

## Abstract

**Objective:**

This *post-hoc* analysis of the EDGE (Effectiveness of Diabetes control with vildaGliptin and vildagliptin/mEtformin) study assessed inter-regional differences in baseline characteristics and response to treatment intensification with dual oral antidiabetes drugs (OADs) in patients with type 2 diabetes mellitus (T2DM).

**Methods:**

Patients with T2DM inadequately controlled with first-line monotherapy were assigned to receive a dipeptidyl peptidase-4 (DPP-4) inhibitor, vildagliptin, or comparator OADs as add-on dual therapy. The primary effectiveness end point (PEP) was achieving glycated hemoglobin (HbA1c) reduction >0.3% without hypoglycemia, peripheral edema, discontinuation owing to gastrointestinal events or weight gain ⩾5% at 12 months. The secondary effectiveness end point (SEP) was achieving HbA1c of <7% without hypoglycemia or weight gain ⩾3% at 12 months.

**Results:**

Baseline characteristics of patients (*N*=43 791), including mean HbA1c (8.2%), varied across regions. Baseline age (62.3 years) and T2DM duration (6.3 years) were greater in patients from Europe than those from India and the Middle East (age: 51.8 and 52.1 years; T2DM duration: 4.3 and 4.2 years, respectively). The probability of achieving PEP with dual therapy was higher in India (odds ratio (OR): 1.5), Latin America (OR: 1.2) and Middle East (OR: 2.0) than in Europe (OR: 0.8) and East Asia (OR: 0.3). Achievement of SEP in patients receiving dual therapy was greater in Latin America (OR: 1.7) and Middle East (OR: 1.7). Vildagliptin add-on therapy allowed more patients to achieve SEP across regions. Women aged ⩾45 years less often attained glycemic target (HbA1c<7%) without significant weight gain ⩾5% compared with women aged <45 years (OR: 0.876, 95% confidence interval: 0.774, 0.992; *P*=0.037).

**Conclusions:**

Baseline HbA1c and T2DM duration differed considerably across all regions. Treatment intensification with second OAD, particularly with a DPP-4 inhibitor vildagliptin, resulted in good treatment response without tolerability issues despite delayed intensification of failing monotherapy across regions.

## Introduction

Type 2 diabetes mellitus (T2DM) is becoming a global health threat owing to its increasing prevalence worldwide.^1, 2^ It is well established that optimal glycemic control prevents the development and progression of macrovascular and microvascular complications of T2DM.^3, 4^

Treatment guidelines recommend a target glycated hemoglobin (HbA1c) of <7% with individualization of therapy based on factors, such as disease duration, patient's life expectancy, vascular complications and other comorbidities.^5, 6^ However, despite the availability of a wide range of therapeutic options and growing awareness regarding disease implications among physicians and patients, approximately 50% of patients with T2DM fail to reach recommended treatment goals.^7^ Because of the progressive nature of T2DM, monotherapy often becomes insufficient to maintain long-term glycemic control,^6^ and therefore, timely intensification of treatment is essential. Nevertheless, as reported earlier, treatment intensification with oral antidiabetes drugs (OADs) has been delayed by approximately 7 years in patients with T2DM.^8^

Data acquired from large real-world observational studies are valuable in gaining insight into the management of disease in diverse clinical health-care settings.^9^ Furthermore, such data help us gain knowledge regarding treatment patterns and patient care across regions and thus would result in better management of T2DM patients worldwide. EDGE (Effectiveness of Diabetes control with vildaGliptin and vildagliptin/mEtformin) was a 1-year, prospective, observational study conducted in patients with T2DM across five regions of the world.^10^

This exploratory *post-hoc* analysis of the EDGE study aimed to assess the existing regional differences worldwide in baseline characteristics and response to dual OADs in patients with T2DM who were inadequately controlled with monotherapy.

## Subjects and methods

### Study design

The EDGE study was conducted at 2957 centers across 27 countries in five regions of the world: Europe, India, the Middle East, Latin America, and East Asia. The details of the study design are presented elsewhere^10^ and are also included in Supplementary Figure S1.

Patients aged ⩾18 years with T2DM who were inadequately controlled on any OAD monotherapy and whose therapy was recently intensified with a second (add-on) OAD were enrolled. The choice of the second OAD was at physicians' discretion based on patients' needs. Patients on any other incretin therapy, those requiring ⩾3 OADs or insulin therapy and those with history of hypersensitivity to any of the study drugs were excluded. In addition, patients were enrolled only after the treatment decision was finalized.

All OADs were prescribed according to country-specific prescription requirements, and all patients were treated as per routine clinical practice. Overall, 45 868 patients were enrolled with documented informed consent, but 2077 had to be excluded because of inadequate source documentation or problems with quality/accuracy of data entry. The intention-to-treat (ITT) population therefore comprised 43 791 patients: 28 442 assigned to the dipeptidyl peptidase-4 (DPP-4) inhibitor, vildagliptin, cohort and 15 349 to the comparator cohort (all other dual OAD combinations excluding incretin-based treatments); 31 patients were not assigned to any cohort.^10^ The protocol for EDGE was approved by local independent review boards or ethics committees. This observational study was designed, implemented and reported in accordance with the International Council for Harmonisation (ICH)-Harmonized Tripartite Guidelines for Good Clinical Practice, where appropriate with applicable local regulations, and with the ethical principles laid down in the Declaration of Helsinki.

### Outcome measures

Baseline demographic characteristics included mean age, body mass index (BMI), duration of T2DM and the most recent HbA1c test results. The change in HbA1c from baseline to the 12-month end point was evaluated in the overall population by world regions.

The primary effectiveness end point (PEP) was the proportion of patients in all the five regions achieving an HbA1c reduction of >0.3% without any tolerability issues, such as peripheral edema, hypoglycemia, discontinuation owing to a gastrointestinal event or a weight gain ⩾5% at 12 months. The secondary effectiveness end point (SEP) included achievement of an HbA1c of <7% at the 12-month end point without a weight gain of ⩾3% or hypoglycemia in patients with a baseline HbA1c of ⩾7% at 12 months. Gender-related differences with respect to treatment intensification, selection of second-line OAD and impact of age on response to dual therapy was assessed and compared between women aged <45 years and ⩾45 years.

Proven hypoglycemia was defined by symptoms suggestive of low plasma glucose levels that resolved promptly upon administration of oral carbohydrates or accompanied by a plasma glucose level <3.1 mmol l^−1^ or any episode requiring the assistance of a third party or hospitalization.

### Statistical analysis

Patient demographics, baseline characteristics and efficacy analyses were described in the ITT population (patients assigned to a new OAD at study initiation). The change in HbA1c (not prespecified in the original study protocol) was adjusted for baseline HbA1c by using an analysis of covariance model and summarized descriptively. For the PEP and SEP, the probability of success was analyzed using a binary logistic regression model to calculate odds ratios (ORs) with 95% confidence intervals (CIs). For each region, the overall ORs for the PEP and SEP were the odds in favor of achieving the end point in the region vs the overall study population or for vildagliptin, in favor of success with comparator OADs. Patients were considered non-evaluable if the outcomes could not be categorized as success or failure owing to missing data for HbA1c or body weight at study end point. These non-evaluable data were considered failures while calculating the OR for success. In this *post-hoc* analysis, only unadjusted ORs are presented. A *P-*value of <0.05 was considered significant. Descriptive statistics and adjusted covariate analysis were performed for gender and age subanalyses. All statistical evaluations were performed using the Statistical Analysis Software, version 9.3 (SAS Institute Inc., Cary, NC, USA).

## Results

### Baseline characteristics

The ITT population included 43 791 patients, of whom, 50.4% (*n*=22 073) were from Europe, followed by India (24.4% *n*=10 692), the Middle East (10.9% *n*=4779), Latin America (8.8% *n*=3846) and East Asia (5.5% *n*=2401). Patient demographics and baseline characteristics markedly varied across the regions and are presented in Table 1.

At baseline, the mean age of patients from Europe was 62.3 years, with a mean T2DM duration of 6.3 years. Patients from India and the Middle East exhibited similar baseline characteristics of mean age (51.8 and 52.1 years, respectively) and T2DM duration (4.3 and 4.2 years, respectively). Overall, 54.8% men were included in the analysis. In India and the Middle East, more men were enrolled (61.4% and 61.6%, respectively). Patients from Europe, the Middle East and Latin America had a higher mean BMI (~30 kg m^−2^) than those from India and East Asia (~26 kg m^−2^).

At initiation of dual therapy, the overall mean baseline HbA1c was 8.2%. The mean baseline HbA1c was higher in India (8.6%), the Middle East and Latin America (both, 8.5%) compared with those in East Asia and Europe (7.7% and 7.9% respectively, Table 1). The incidences of macrovascular and microvascular complications at baseline in the vildagliptin cohort (*n*=28 442) was 13.1% and 7.4% respectively; in the comparator cohort (*n*=15 349) the incidence was 11.0% and 7.3% for macrovascular and microvascular complications, respectively. Patients from Europe had the highest incidence of macrovascular (18.3% vildagliptin cohort, 18.6% comparator cohort) and microvascular complications (9.3% vildagliptin cohort, 10.3% comparator cohort) as compared with other regions (Supplementary Figure S2).

**Figure 1:**
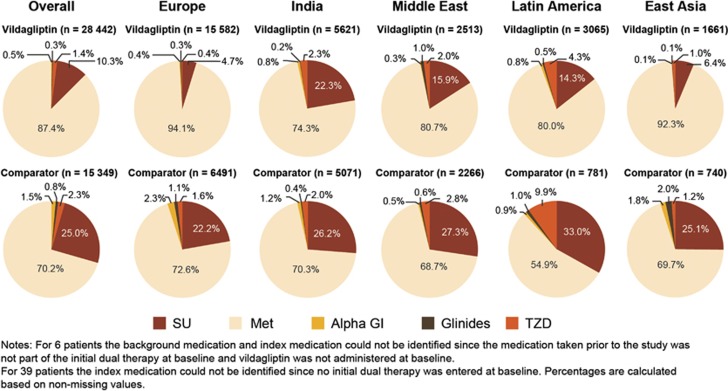
Background OAD therapy across five regions at study entry by cohort (ITT population). Alpha GI, alpha-glucosidase inhibitors; ITT, intention-to-treat; Met, metformin; OAD, oral antidiabetes drugs; TZD, thiazolidinediones.

At the time of treatment intensification (addition of a second OAD), most patients received metformin in both the vildagliptin (87.4%) and comparator (70.2%) cohorts, followed by sulfonylureas (SUs; vildagliptin: 10.3% comparator: 25.0%) as a first-line monotherapy (Figure 1).

### Primary and secondary effectiveness end points

Overall, patients with higher baseline HbA1c showed greater HbA1c reduction at the 12-month end point. The odds of successfully achieving the PEP with dual therapy were higher in patients from India (OR: 1.5), Latin America (OR: 1.2), and the Middle East (OR: 2.0) compared with those in Europe (OR: 0.8) and East Asia (OR: 0.3). The proportion of non-evaluable patients was higher in East Asia (48.7%) compared with other regions (Table 2). The odds of successfully achieving the SEP of an HbA1c of <7.0% without hypoglycemia or weight gain were the highest in Latin America and the Middle East (both, OR: 1.7) and the lowest in East Asia (OR: 0.5, Table 2).

**Figure 2:**
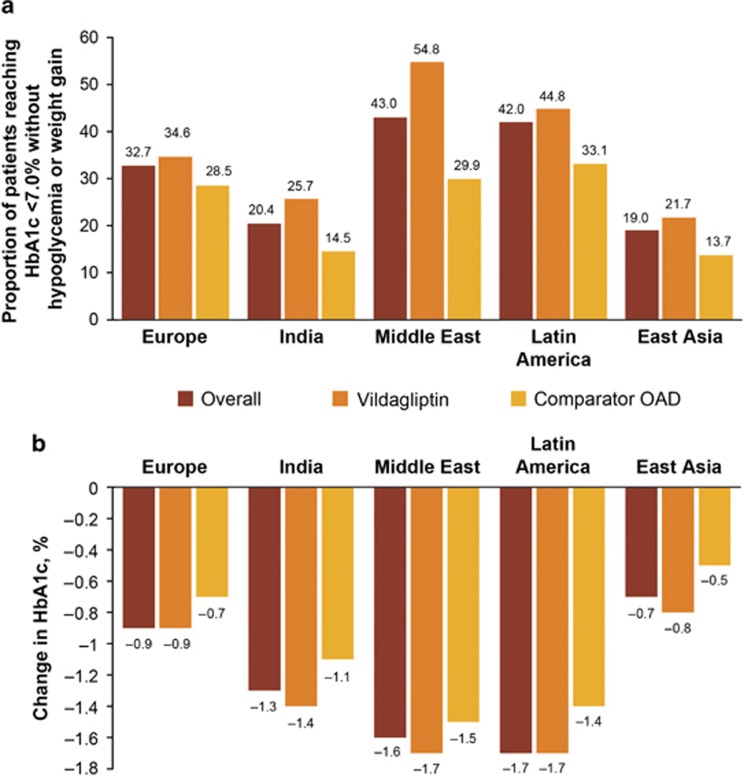
(**a**) Proportion of patients achieving glycemic goal* after 1 year of treatment by region. (**b**) Mean change in HbA1c after 1 year of treatment by region (ITT population). Overall data are taken from ITT population and cohort data are taken from PP population. *HbA1c<7.0% without hypoglycemia or weight gain ⩾3% in patients with baseline HbA1c ⩾7.0% (SEP). HbA1c, glycated hemoglobin; ITT, intention-to treat; OAD, oral antidiabetes drug; PP, per protocol; SEP, secondary effectiveness end point.

Similarly, the proportion of patients achieving the SEP (Figure 2a) and the mean HbA1c reduction from baseline to end point (Figure 2b) were higher in the Middle East and Latin America. The overall HbA1c reduction from baseline was higher in the Middle East (−1.6%) and Latin America (−1.7%) compared with that in Europe (−0.9%), East Asia (−0.7%) and India (−1.3%). Within each region, both the proportion of patients achieving the SEP and the mean HbA1c reduction were higher in the vildagliptin cohort than in the comparator cohort (Figures 2a and b).

### Gender and age subanalyses

Treatment intensification occurred marginally earlier in women (8.1±1.33% (mean±s.d.) vs men: 8.2±1.34% difference: 0.113% 95% CI: 0.087, 0.139; *P*<0.001). Furthermore, this intensification with second-line therapy occurred later in women <45 years of age (*n*=2072; HbA1c: 8.3±1.32%) vs women aged ⩾45 years (*n*=17 728; HbA1c: 8.1±1.32%). In many regions, women <45 years of age had high HbA1c and already manifested with macrovascular and microvascular complications despite a shorter T2DM duration at study entry (3.1±3.32 vs 6.2±5.52 years).

The second-line treatment option varied across regions and gender. Particularly in India and the Middle East, men were prescribed dual therapy with SUs or DPP-4 inhibitor more often compared with women; more men received DPP-4 inhibitor containing dual therapy (Middle East: 36% women vs 64% men; India: 38% women vs 62% men). In the other regions, dual therapy regimen was relatively more equal in distribution among men and women.

Overall, women aged ⩾45 years less often attained glycemic target (HbA1c<7%) without a significant weight gain of ⩾5% compared with women aged <45 years (OR: 0.876, 95% CI: 0.774, 0.992; *P*=0.037). Despite this, the mean end-of-study HbA1c was similar for women irrespective of age (7.03% vs 7.08%, respectively).

## Discussion

This *post-hoc* analysis from the EDGE study showed marked differences in baseline characteristics of patients with T2DM across the regions.

At baseline, patients in Europe were approximately 10 years older (mean age, 62.3 years) with a longer T2DM duration (6.3 years) than patients in India (51.8 and 4.3 years, respectively) or the Middle East (52.1 and 4.2 years, respectively). This difference in the baseline age and T2DM duration suggests early onset of T2DM in developing countries that could be attributed to the variations in lifestyle, urbanization, physical activity and dietary habits.^11, 12^ In addition, increased prevalence of impaired glucose tolerance, abdominal adiposity and insulin resistance also adds to the risk of developing T2DM at a younger age in these regions.^12, 13^ More men were included in India (61.4%) and the Middle East (61.6%).

Although it is well known that several chronic complications can occur owing to poor glycemic control,^14, 15, 16^ the mean HbA1c at baseline in the present study was 8.2%,^10^ suggesting the presence of suboptimal glycemic control worldwide. In East Asia and Europe, physicians prescribed the second OAD at a baseline HbA1c of 7.7% and 7.9%, respectively, compared with other regions, where physicians prescribed the second OAD at an HbA1c of approximately 8.6%. There was a general trend of delay in treatment intensification across all the regions, although the extent of this delay was variable.

Guidelines recommend early treatment intensification to reach the target HbA1c, particularly in patients with shorter disease duration and longer life expectancy,^6, 8^ but patients from India and the Middle East exhibited high baseline HbA1c at the time of addition of a second OAD. This lack of appropriate treatment intensification for T2DM, particularly in low- and middle-income countries, may be attributed to poor health-care infrastructure, high treatment and consultation costs, low adherence to treatment modification guidelines and the preference for traditional or alternative forms of treatment.^17, 18, 19^ Additionally, the presence of clinical inertia could also contribute to the poor glycemic control seen in the present study.^4, 20^ At baseline, the incidences of macrovascular and microvascular complications were higher in patients from Europe compared with patients from other regions. Older age, longer disease duration and higher BMI may have been the predisposing factors for development of macrovascular and microvascular complications in patients from Europe.

With respect to achievement of the PEP, the probability of success was higher in India, Latin America and the Middle East compared with Europe and East Asia, and the adjusted mean change in HbA1c from baseline to the study end point was high. This finding is consistent with previous reports that showed that higher baseline HbA1c results in greater reductions after treatment.^21^ The observed differences in the mean change in HbA1c may be explained by factors, such as baseline HbA1c, duration of T2DM, insulin sensitivity, insulin resistance, baseline BMI, access to and intensity of care and genetic and ethnic variations that may affect response to treatment.^22, 23, 24, 25^ Further analysis by treatment cohort with respect to the PEP showed that dual combination with vildagliptin showed better effectiveness and tolerability than any other dual OAD combinations. These results were consistent with the efficacy and tolerability profile of vildagliptin reported in other randomized controlled trials (RCTs).^26, 27, 28, 29, 30^

The SEP was achieved in a higher proportion of patients in Latin America and the Middle East compared with that in the other regions, irrespective of the dual therapy used. Similarly, addition of vildagliptin as a second OAD showed clinically meaningful differences from the comparator cohort in achieving the clinically important SEP (HbA1c<7% without hypoglycemia or a weight gain ⩾3%) in patients with baseline HbA1c of ⩾7% across all the regions after 12 months. Moreover, other DPP-4 inhibitors have showed favorable effects in terms of HbA1c goal attainment when used as an add-on therapy in a retrospective analysis of the real-world data and in a pooled analysis of RCTs.^14, 31^

Vildagliptin dual combination therapy has demonstrated consistent effectiveness in real-world studies comparable to RCTs, whereas other OADs, particularly SUs, have shown increasingly reduced effectiveness and blunting of the baseline-dependent glycemic response in real-world than in RCTs especially when approaching the normoglycemic range; this disparity may be attributed to suboptimal use of OADs, such as SUs, owing to fear of hypoglycemia or weight gain.^32^

Gender differences in choice of second-line therapy and evidence of delayed intensification of treatment in women aged <45 years who already presented with well-established diabetes complications suggest that further action is warranted to alleviate the impact of patients' and physicians' attitude and also influences of culture and environmental factors on the treatment of T2DM worldwide.

### Strengths and limitations

EDGE was one of the largest prospective, observational studies of any incretin-based therapy that enrolled patients from five world regions.^10^ The findings of this study are reflective of global real-world diabetes care involving physicians and health-care settings from a vast spectrum of practices.

The choice of treatment was at physician's discretion, which was intended to negate inclusion bias in recruitment. On the contrary, this could have also been a potential cause for bias owing to non-uniformity in recruitment across the regions. The higher proportion of patients in the vildagliptin cohort can be explained by the fact that physicians may have chosen to prescribe a relatively new drug with a better safety and tolerability profile compared with other OADs. Moreover, EDGE was conducted at the time when DPP-4 inhibitors were only newly added to the treatment algorithm, which would have raised expectations and interest among physicians to test the effectiveness of DPP-4 inhibitors, such as vildagliptin. This has been further reiterated in a recent retrospective study that showed the changing trends in the selection of second-line OADs, with an increased use of incretin therapy vs a decreased use of thiazolidinediones and SUs over the past decade.^33^ Like any other observational study, our study had certain limitations, such as lack of randomization and centralized laboratory settings, which may have led to variability in measurements and regular safety monitoring.

## Conclusion

This *post-hoc* analysis showed that baseline HbA1c and duration of T2DM differs considerably across regions of the world. The presence of suboptimal glycemic control across world regions suggests the need for early treatment intensification. Educational programs for physicians, patient motivation, intensive monitoring of glycemic control, better patient–physician communication and timely intensification of therapy would help prevent long-term complications associated with T2DM.

Despite delay in intensification of therapy, a favorable HbA1c response was observed following addition of a second OAD across all the regions. Vildagliptin dual combination allowed more patients to achieve clinically relevant HbA1c reduction without tolerability issues, such as hypoglycemia or weight gain.

## Figures and Tables

**Table 1:** Patient demographics and baseline characteristics (ITT population)

*Parameter*	*Overall*, N*=43 791*	*Europe*, n*=22 073*	*India*, n*=10 692*	*Middle East*, n*=4779*	*Latin America*, n*=3846*	*East Asia,* n*=2401*
Age, years	57.8±11.78	62.3±10.89	51.8±9.95	52.1±10.23	55.9±12.41	57.2±11.40
Men, *n* (%)	23 990 (54.8)	11 488 (52.0)	6561 (61.4)	2942 (61.6)	1823 (47.4)	1176 (49.0)
Women, *n* (%)	19 801 (45.2)	10 585 (48.0)	4131 (38.6)	1837 (38.4)	2023 (52.6)	1225 (51.0)
BMI, kg m^−2^	29.0±5.14	30.3±5.22	26.6±4.06	29.4±4.71	29.5±5.31	25.2±3.38
HbA1c,^a^ %	8.2±1.32	7.9±1.27	8.6±1.11	8.5±1.27	8.5±1.70	7.7±1.30
Duration of T2DM, years	5.5±5.25	6.3±5.61	4.3±4.11	4.2±3.98	5.7±6.12	5.7±5.45

Abbreviations: BMI, body mass index; HbA1c, glycated hemoglobin; ITT, intention-to-treat; T2DM, type 2 diabetes mellitus. Data are expressed as mean±s.d., unless specified otherwise.

a HbA1c value based on which the second-line oral antidiabetes drug was added/decided upon.

**Table 2:** Primary and secondary overall effectiveness end points by regions (ITT population)

*Parameter*	*Overall,* N*=43 791*	*Europe*, n*=22 073*	*India*, n*=10 692*	*Middle East*, n*=4779*	*Latin America,* n*=3846*	*East Asia,* n*=2401*
*Primary effectiveness end point*^a^						
Success rate	23 533 (53.7)	10 642 (48.2)	6738 (63.0)	3301 (69.1)	2229 (58.0)	623 (26.0)
Non-evaluable	11 395 (26.0)	5877 (26.6)	2442 (22.8)	858 (18.0)	1048 (27.3)	1170 (48.7)
OR (95% CI)	1	0.8 (0.81, 0.87)	1.5 (1.47, 1.59)	2.0 (1.90, 2.12)	1.2 (1.17, 1.31)	0.3 (0.29, 0.34)
						
*Secondary effectiveness end point*^b^						
Success rate	11 040 (30.8)	5498 (32.7)	2002 (20.4)	1922 (43.0)	1306 (42.0)	312 (19.0)
Non-evaluable	6897 (19.2)	3754 (22.3)	1158 (11.8)	631 (14.1)	608 (19.6)	746 (45.5)
OR (95% CI)	1	1.1 (1.07, 1.16)	0.6 (0.56, 0.62)	1.7 (1.63, 1.82)	1.7 (1.56, 1.77)	0.5 (0.49, 0.59)

Abbreviations: CI, confidence interval; ITT, intention-to-treat; OR, odds ratio.

a The proportion of patients in all the five regions achieving a glycated hemoglobin (HbA1c) reduction of >0.3% without any tolerability issues, such as peripheral edema, hypoglycemia, discontinuation owing to a gastrointestinal event or a weight gain of ⩾5% at 12 months.

b In patients with a baseline HbA1c of ⩾7.0%, achievement of the target HbA1c of <7.0% at the 12-month end point, without a weight gain of ⩾3% at 12 months or hypoglycemic event.
