# Supplementary figures and images for: Differences in glycemic control across world regions: a post-hoc analysis in patients with type 2 diabetes mellitus on dual antidiabetes drug therapy
Source: Nutr Diabetes. 2016 Jul 4;6(7):e217–. doi: 10.1038/nutd.2016.25 (PMC4973138; doi:10.1038/nutd.2016.25)

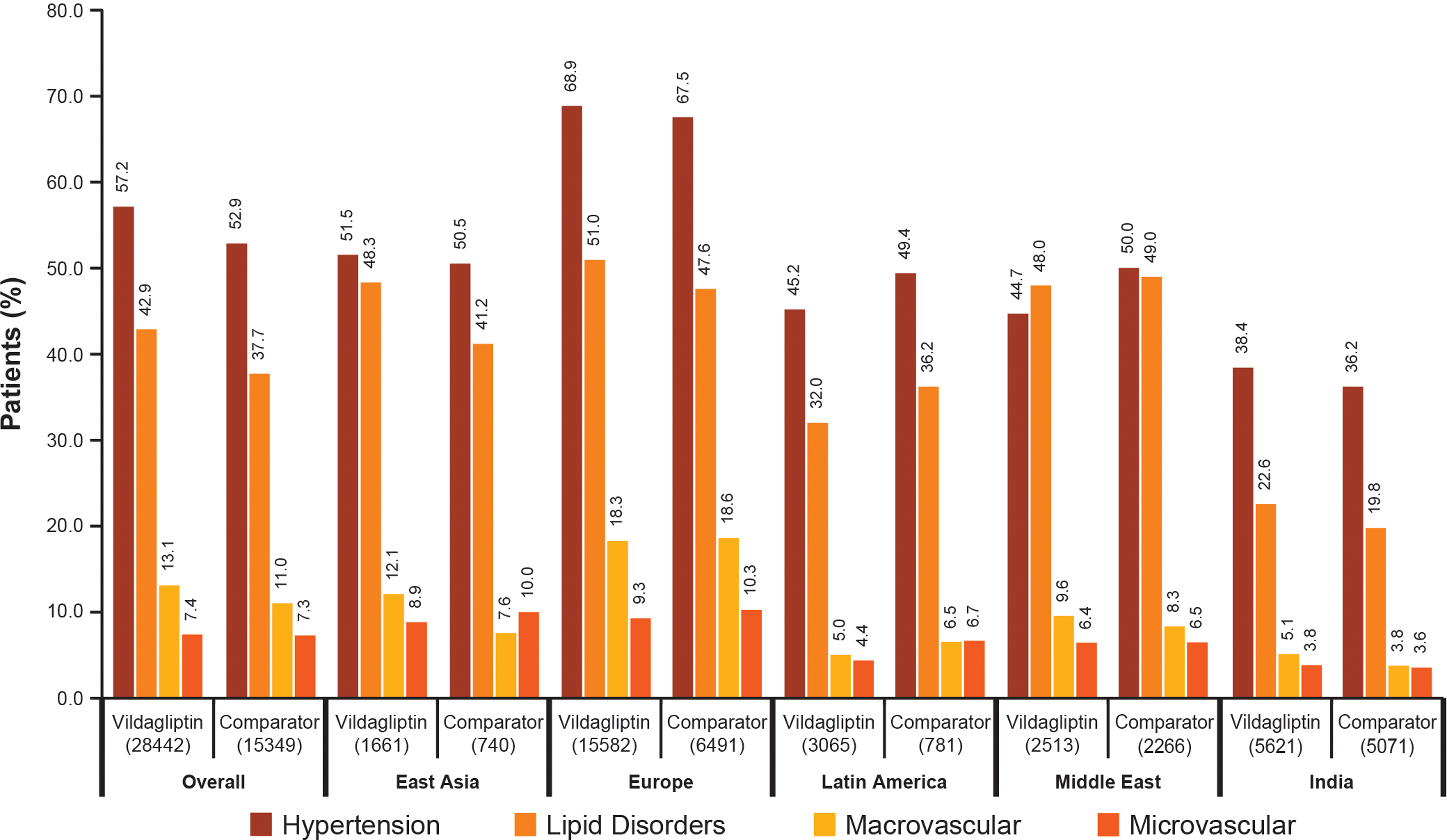

Supplement: Supplementary Figure 2 [file nutd201625x2.tif]
