# Supplementary material for: Differences in glycemic control across world regions: a post-hoc analysis in patients with type 2 diabetes mellitus on dual antidiabetes drug therapy
Source: Nutr Diabetes. 2016 Jul 4;6(7):e217–. doi: 10.1038/nutd.2016.25 (PMC4973138; doi:10.1038/nutd.2016.25)
Supplement: Supplementary Information [file nutd201625x3.doc]

**Supplementary section:**

The countries included from the five regions were as follows:

- Latin America: Argentina, Colombia, Ecuador, Mexico, Venezuela
- Europe: Austria, Belgium, Bulgaria, the Czech Republic, Germany, Greece, Luxembourg, the Netherlands, Portugal, the Russian Federation, Slovakia, Sweden
- India
- East Asia: The Philippines, the Republic of Korea
- Middle East: Bahrain, Jordan, Kuwait, Lebanon, Oman, Palestine, the United Arab Emirates

Drawbacks of study design:

- Observational studies are limited by the lack of randomization, with a recognized risk of bias and confounding.
- Treatment choice was made by the physician; this could be affected by multiple factors that cannot be statistically adjusted, and may hence not allow for a well-balanced patient population.
- Incretin-based drugs other than vildagliptin were not used in the study to avoid confounding the comparison between incretin therapies and other OADs.
- Recruitment bias cannot be ruled out in terms of differences in the healthcare setups that enrolled patients.
